# Retraction Note: An autocatalytic multicomponent DNAzyme nanomachine for tumor-specific photothermal therapy sensitization in pancreatic cancer

**DOI:** 10.1038/s41467-026-75939-0

**Published:** 2026-08-03

**Authors:** Jiaqi Yan, Xiaodong Ma, Danna Liang, Meixin Ran, Dongdong Zheng, Xiaodong Chen, Shichong Zhou, Weijian Sun, Xian Shen, Hongbo Zhang

**Affiliations:** 1 Joint Centre of Translational Medicine, Wenzhou Key Laboratory of Interdiscipline and Translational Medicine, The First Affiliated Hospital of Wenzhou Medical University, Wenzhou, China; 2 Department of General Surgery, The First Affiliated Hospital of Wenzhou Medical University, Wenzhou, Zhejiang China; 3 Pharmaceutical Sciences Laboratory, Faculty of Science and Engineering, Åbo Akademi University, Turku, Finland; 4 Turku Bioscience Centre, University of Turku and Åbo Akademi University, Turku, Finland; 5 Department of Gastrointestinal Surgery, The Second Affiliated Hospital and Yuying Children’s Hospital of Wenzhou Medical University, Wenzhou, 325027 Zhejiang China; 6 Department of Ultrasound, Fudan University Shanghai Cancer Center, Shanghai, 200032 PR China

Retraction Note: *Nature Communications* 10.1038/s41467-023-42740-2, published online 30 October 2023

The authors have retracted this article. After publication, the authors identified the following errors in the figures:

- Fig. 8a duplicated 24 h and 48 h images;
- Figure 8g, 9c, and 9g image errors and mislabelling;
- Figure 8h and 9d incorrect data in the plots.

Further checks by the publisher have found that Figure 8c 0 Day I + C + P mice 1, 2 and 3 appear highly similar.

The authors have provided the underlying raw data and stated that these errors were introduced during figure preparation. Given the number of these errors, the authors have decided to retract the article.

All authors agree with this retraction.

